# Prediction models for fear of cancer recurrence in adults with cancer: a systematic review

**DOI:** 10.3389/fonc.2026.1739251

**Published:** 2026-03-19

**Authors:** Jiazheng Wang, Hengjin Wu, Tongtong Xu, Zhentao Lu, Haoran Chen, Xudong Zhu, Yilin Wang, Hao Liu

**Affiliations:** 1 Guang’anmen Hospital, China Academy of Chinese Medical Sciences, Beijing, China; 2 The 80th Group Army Hospital, People’s Liberation Army, Weifang, Shandong, China; 3 Affiliated Hospital of Traditional Chinese Medicine, Guangzhou Medical University, Guangzhou, China; 4 Graduate School of Beijing University of Traditional Chinese Medicine, Beijing, China

**Keywords:** cancer, fear of cancer recurrence, modelling, prediction models, system review

## Abstract

**Background:**

Numerous studies have developed or validated prediction models to estimate the likelihood of Fear of Cancer Recurrence (FCR) among patients with cancer. The quality of these models and evaluations of their applicability to clinical practice and future research remain unclear. This study systematically evaluated the risk of bias and applicability of prediction models for FCR in oncology populations.

**Methods:**

We searched PubMed, Embase, Web of Science, the Cochrane Library, Cumulative Index to Nursing and Allied Health Literature (CINAHL), China National Knowledge Infrastructure (CNKI), China Science and Technology Journal Database (VIP), Wanfang Data, and Chinese Biomedical Literature Database (CBM) from inception to August 1, 2025. Two reviewers independently screened studies and extracted data. We used the Prediction model Risk Of Bias ASsessment Tool (PROBAST) checklist to assess risk of bias and applicability.

**Results:**

We identified 702 records and ultimately included 7 studies encompassing 18 models. Most were published between 2022 and 2025. Sample sizes in the included studies ranged from 200 to 918, and the reported discrimination in model development cohorts ranged from 0.660 to 0.996. Applicability was judged to be good across all studies. However, all studies exhibited a high risk of bias, mainly due to suboptimal data sources, inadequate handling of predictors and missing data, and limited model validation. Among the included studies, the prevalence of FCR ranged from 47.7% to 63.4%. The most frequent predictors were age, social support, household/per-capita monthly income, occupation or employment status, and fatigue.

**Conclusions:**

Prediction modeling for FCR in patients with cancer remains in an early stage, with both shared and heterogeneous predictors. Although overall model performance appears acceptable, most studies have methodological shortcomings, and few models have been validated. Future studies should design, conduct, and report models in accordance with the Transparent Reporting of a multivariable prediction model for Individual Prognosis Or Diagnosis (TRIPOD) guideline. In addition, studies with larger sample sizes and multicenter external validation are needed to improve model robustness.

## 1 Introduction

With global population aging and rapid advances in cancer diagnosis and treatment, the worldwide cancer burden continues to rise; GLOBOCAN 2022 estimates about 20 million new cases and 9.7 million deaths in 2022 (1). The World Health Organization (WHO) Cancer Report 2024 indicates that the number of cancer survivors worldwide has exceeded 50 million (2). Cancer is characterized by a prolonged disease course, high medical costs, and therapeutic complexity; diagnosis and treatment expose patients to physiological, emotional, and social pressures, imposing substantial economic strain on individuals and health systems (3). For patients with cancer, the risk of recurrence spans the entire continuum of care—from diagnosis and treatment to rehabilitation and follow-up—and is a key determinant of long-term quality of life and health care utilization (4). Fear of Cancer Recurrence (FCR) is defined as “a persistent fear, worry, or anxiety regarding the possibility of cancer returning or progressing” (5) and has repeatedly been shown to be one of the most prevalent and persistent psychological concerns among cancer survivors; more than half of patients with cancer report moderate levels of FCR (6). Studies report a 20%–30% increase in health service use among individuals with high FCR (7). At the same time, FCR remains common in long-term survivors and does not markedly diminish over time, with high prevalence even 8 years after diagnosis. In the absence of targeted intervention, its impact on quality of life and health systems is long-lasting and far-reaching (8).

An international multicenter Delphi study further clarifies the core clinical features of FCR: hypervigilance to signs of recurrence (e.g., misinterpreting ordinary fatigue as a sign of relapse), uncontrollable repetitive worry, excessive preoccupation with disease-related information, and fear-driven avoidance behaviors (e.g., declining follow-up examinations) (9). These features not only affect psychological well-being but may also indirectly influence cancer outcomes by reducing treatment adherence and disrupting healthy lifestyle choices. However, current research on FCR has limitations. Most studies (10–12) focus on estimating prevalence and examining associated factors (e.g., higher risk among women, younger patients, and those with pain or fatigue), yet findings show marked heterogeneity. A systematic review by Simard et al. (13) reports a 22%–87% prevalence of moderate-to-high FCR among patients with cancer. This spread reflects non-standardized assessment tools—such as the Fear of Cancer Recurrence Inventory (FCRI) and the Fear of Progression Questionnaire–Short Form (FoP-Q-SF)—and a lack of consensus on cutoff thresholds across studies (e.g., FCRI ≥13 vs ≥22). Predictive research on FCR also lags behind, limiting early identification of high-risk patients in clinical practice.

In current clinical practice, clinicians often rely on subjective judgment to assess the risk of FCR, and objective, usable prediction tools are lacking, resulting in delayed intervention for some high-risk patients. To better enable precision supportive care and psychological interventions, multivariable clinical prediction models can facilitate early identification of patients at high risk of FCR, thereby supporting risk stratification and individualized intervention. Accordingly, this review will systematically search the global literature on FCR prediction models and provide a comprehensive synthesis of model construction approaches (e.g., logistic regression and machine learning algorithms), predictor domains (demographic, clinical, and psychosocial factors), performance metrics (e.g., area under the curve [AUC], sensitivity, specificity, and calibration), and validation practices, and will analyze the strengths and limitations of existing models. On the one hand, it will offer clinical researchers an overview of existing FCR prediction models and clarify directions for optimization (e.g., adding missing predictors, adopting more suitable algorithms, and strengthening external validation). On the other hand, it will help clinicians identify prediction tools with higher practical utility to enable early recognition and targeted intervention for patients at high risk of FCR, ultimately shifting FCR management from reactive treatment to proactive prevention and improving long-term psychological outcomes and quality of life in patients with cancer.

## 2 Methods

### 2.1 Search strategy

To ensure comprehensive coverage, we targeted both Chinese- and English-language databases. We searched nine databases—PubMed, Embase, Web of Science, the Cochrane Library, Cumulative Index to Nursing and Allied Health Literature (CINAHL), China National Knowledge Infrastructure (CNKI), China Science and Technology Journal Database (VIP), Wanfang Data, and Chinese Biomedical Literature Database (CBM)—from inception to August 1, 2025. We combined subject-heading and free-text searches to identify literature on prediction models for FCR in patients with cancer; Search keywords included “tumor”, “cancer”, “carcinoma”, “fear of recurrence”, “fear of cancer recurrence”, “fear of tumor recurrence”, “prediction”, “model”, “prediction model”, “risk prediction”, “risk assessment”, and “risk factors”. We also screened the reference lists of retrieved studies and reviews to identify additional records. The full search strategies are provided in Appendix 1 to enhance reproducibility.

In this systematic review, we used the PICOTS framework recommended in the Checklist for critical Appraisal and data extraction for systematic Reviews of prediction Modelling Studies (CHARMS) (14). This approach helps specify the review objectives, search strategy, and inclusion/exclusion criteria (15). The core components of our review were as follows:

P (Population): Adults (≥18 years) diagnosed with cancer, regardless of tumor type or treatment phase.
I (Index prediction model): Development and/or validation of prediction models for the risk of FCR in patients with cancer.
C (Comparator): Not applicable.
O (Outcome): FCR occurrence or high risk as defined by validated instruments.
T (Timing): No restrictions; both short- and long-term prediction horizons were eligible.
S (Setting): Oncology care and follow-up settings; intended for early clinical identification and risk stratification to guide interventions.

### 2.2 Inclusion and exclusion criteria

Inclusion criteria:

1. Participants were patients with cancer aged ≥18 years.
2. Studies focused on the development and/or validation of risk prediction models, with FCR as the primary outcome.
3. Eligible designs included case–control, cross-sectional, and cohort studies.
4. Publications in Chinese or English.

Exclusion criteria:

1. Studies that analyzed risk factors only without constructing a complete prediction model.
2. Studies in which the final prediction model contained fewer than two predictors.
3. Conference papers and review articles.
4. Duplicate publications, studies with incomplete data, or records without full-text availability.

### 2.3 Study selection

Retrieved records were imported into reference management software. First, duplicate studies were identified and removed manually. Second, titles and abstracts were screened. Finally, full texts were assessed after applying the inclusion and exclusion criteria. Reference lists of all eligible studies were also checked to ensure comprehensive retrieval. Two reviewers performed study selection independently and cross-checked the results. Disagreements were resolved through discussion with a third reviewer.

### 2.4 Data extraction

After study selection, data were extracted from eligible studies using the CHARMS checklist (14). Extracted items included population, study design, data source, sample size, missing data, predictors, model development and validation methods, and model performance. Two reviewers independently performed and cross-checked data extraction. Any discrepancies were resolved with input from a third reviewer.

### 2.5 Risk of bias and applicability assessment

We assessed risk of bias and applicability using the Prediction model Risk Of Bias ASsessment Tool (PROBAST) (16). Risk of bias was evaluated across 20 signaling questions within four domains: participants, predictors, outcome, and analysis. Applicability was evaluated across three domains: participants, predictors, and outcome. Overall risk of bias was rated “low” only when all domains were judged low risk; it was rated “high” when ≥1 domain was judged high risk; and it was rated “unclear” when one domain was unclear and all remaining domains were low risk. Overall applicability was judged using analogous rules. Two reviewers conducted the assessments independently and cross-checked each other’s ratings. Disagreements were resolved by discussion, with arbitration by a third reviewer when needed.

### 2.6 Descriptive analysis

We summarized the included studies, model development, and model performance using descriptive statistics.

### 2.7 Patient and public involvement

No patients or members of the public were involved in this study.

## 3 Results

### 3.1 Study selection process

**Figure 1:**
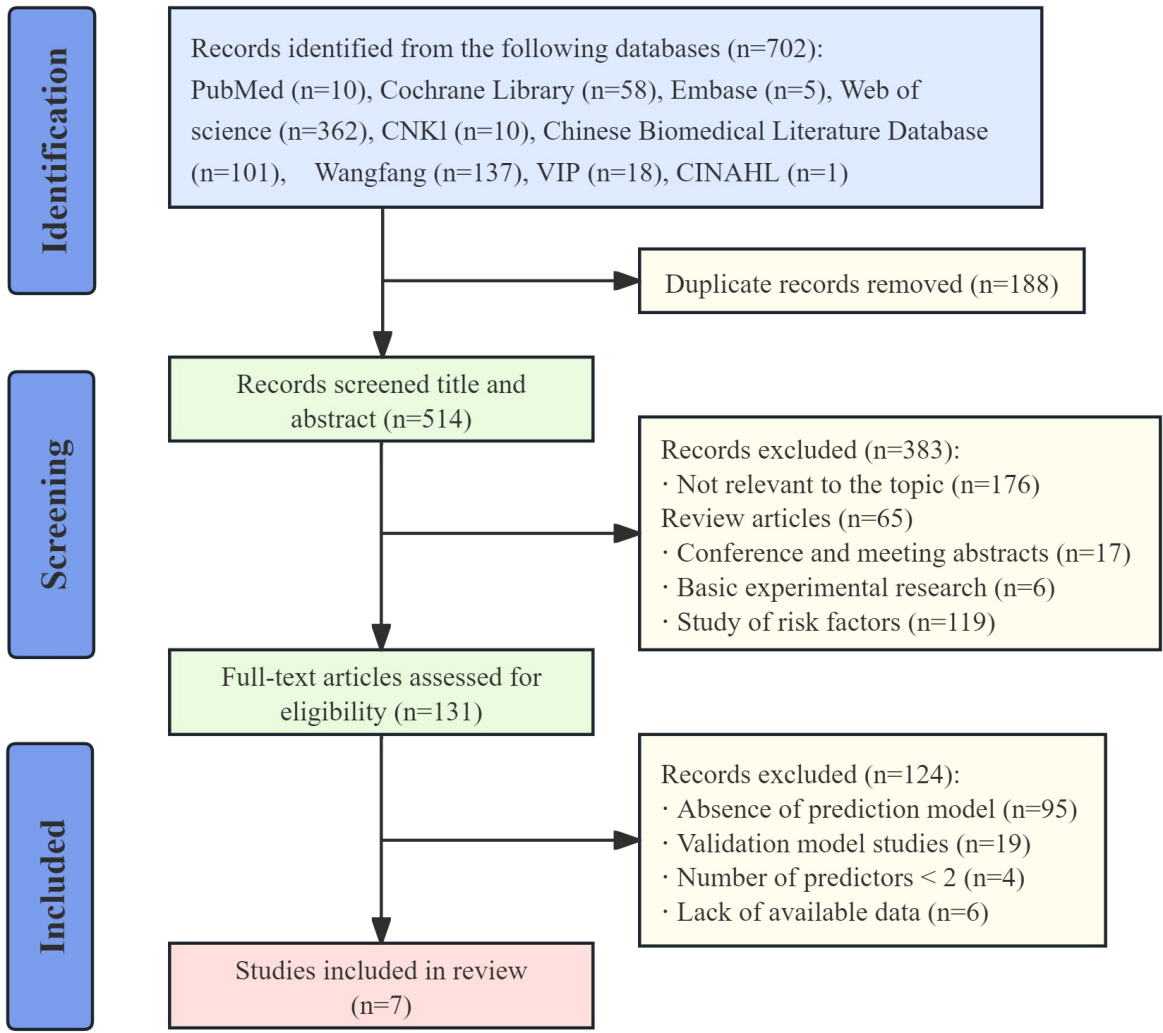
Process of study selection.

The search identified 702 records, of which 188 duplicates were removed. After title and abstract screening, 383 records were excluded. Full-text assessment led to a further exclusion of 124 records (95 articles lacked a prediction model, 19 were validation-only model studies, 4 included fewer than two predictors in the final model, and 6 had insufficient data). In total, 7 studies (17–23) were included in this systematic review. The study selection process is presented in **Figure 1**.

### 3.2 Study characteristics

This review included seven studies. These studies were published between 2022 and 2025. Study populations covered breast cancer (newly diagnosed, rehabilitation phase, and survivors), postoperative thyroid cancer, postoperative non-small-cell lung cancer, and gastrointestinal tumors. Among the included studies, six were conducted in China (17–21, 23) and one was conducted in France (22). Regarding study design, five were cross-sectional studies (17, 19–21, 23), one was a retrospective cohort study (22), and one was a case-control study (18). One study was a nationwide, machine-learning-based methodological study (22); the remainder were mostly single-center, with one multicenter study (23). Primary outcomes were mainly defined using the Fear of Cancer Recurrence Inventory-Short Form (FCRI-SF) or the Fear of Progression Questionnaire-Short Form (FoP-Q-SF). Detailed characteristics of the included studies are summarized in **Table 1**.

**Table 1:** Basic characteristics of inclusion in the literature.

Author(s)(year)	Region	Study design	Participants	FCR ascertainment	FCR prevalence (%)
Man Liu et al.(2024) (20)	China	Cross-sectional study	NSCLC postoperative patients	FoP-Q-SF	Not reported
Yaru Li et al.(2024) (21)	China	Cross-sectional study	GI cancer patients	FCRI-SF	47.70%
Mamoudou Koume et al.(2024) (22)	France	Retrospective cohort study	Breast cancer patients	Patient self-report	63.40%
Hui Ren et al.(2024) (23)	China	Cross-sectional study	Breast cancer postoperative patients	FCRI-SF	58.80%
Linlin Li et al.(2025) (18)	China	Case-control study	Thyroid cancer postoperative patients	FoP-Q-SF	Not reported
Chao Fang et al.(2025) (17)	China	Cross-sectional study	Breast cancer patients	FoP-Q-SF	Not reported
Huiying Yang (2022) (19)	China	Cross-sectional study	Breast cancer patients	FoP-Q-SF	Not reported

NSCLC, non-small cell lung cancer; GI, gastrointestinal; FoP-Q-SF, Fear of Progression Questionnaire–Short Form; FCRI-SF, Fear of Cancer Recurrence Inventory–Short Form.

### 3.3 Basic characteristics of prediction models

Among the included studies, three studies (18, 21, 23) developed a single model, whereas four studies (17, 19, 20, 22) developed multiple models, yielding a total of 18 models. Notably, the studies by Mamoudou Koume et al. (22) and Chao Fang et al. (17) contributed five models each. In the end, our systematic review evaluated 11 models. Across studies, sample sizes ranged from 200 to 918, and none of the included studies reported missing sample cases. For model development, most studies used logistic regression. In addition, one study (20) employed a backpropagation neural network (BPNN), and two studies (17, 22) developed prediction models using machine-learning techniques. Regarding predictors, the final number per model ranged from 5 to 12. The five most frequent predictors were age, social support, household/per-capita monthly income, occupation/employment status, and fatigue. Basic characteristics of the prediction models are summarized in **Table 2**.

**Table 2:** Overview of the information of the included prediction models.

Author(s)(year)	Modeling methods (number of models)	Candidate variables		Sample size (development cohorts/validation cohorts)				Final predictors	Model presentation	Selection of variables
		Number	Continuous variable processing	Total sample size	Build	Verify	Missing data			
Man Liu et al.(2024) (20)	LR, BPNN (2)	9	Continuous variables were left unaltered	596	427	169	–	Age, gender,levels of education,residenc,monthly income,whether children are underage,hope index, social support	Backpropagation neural network	Multifactor analysis
Yaru Li et al.(2024) (21)	LR (1)	6	categorical coding	570	–	–	–	Gender,therapy,alimentary tract haemorrhage,pain, depression,social support	Nomogram	Multifactor analysis
Mamoudou Koume et al.(2024) (22)	RF, SVM, GB, XGBoost, MLP (5)	20	Continuous variables were left unaltered	918	–	–	–	Echography,spinal column radiography,radiology, breast MRI,imaging procedures of the musculoskeletal system,drugs related to acidity problems,benzodiazepine derivatives, broad-spectrum penicillins,NSAIDs,Vitamin D and analogues,standard blood biochemistry, standard hormone assays	–	Feature selection and oversampling methods
Hui Ren et al.(2024) (23)	LR (1)	21	Continuous variables were left unaltered	781	548	233	–	Fatigue,social constraints,maladaptive cognitive emotional regulation strategies,meta-cognition,age	Nomogram	Multifactor analysis
Linlin Li et al.(2025) (18)	LR (1)	5	Continuous variables were left unaltered	200	–	–	–	Age,family per capita monthly income,rapid rehabilitation care,social support rating scale score, fear of progression questionnaire short form score	Nomogram	Multifactor analysis
Chao Fang et al.(2025) (17)	XGBoost, LightGBM, DT, RF, MLR(5)	8	Continuous variables were left unaltered	339	250	89	–	Age,years of marriage,occupation,employment status,monthly per capita household income,level of social support,coping strategies (facing and avoiding)	–	Single-factor and correlation analyses
Huiying Yang (2022) (19)	LR, ANN, RF (3)	28	Continuous variables were discretized	490	–	–	–	Age,occupational status,chemotherapy,self-image, cancer-related fatigue,disease perception	–	Multifactor analysis

LR, logistic regression;BPNN, Back propagation Neural Network; MLR, multiple logistic regression; DT, decision tree; RF, random forest; SVM, support vector machine; GB, gradient boosting; XGBoost, extreme gradient boosting; LightGBM, Light Gradient Boosting Machine; MLP, multilayer perceptron; ANN, artificial neural network; MRI, magnetic resonance imaging; NSAIDs, non-steroidal anti-inflammatory drugs; -,not reported.

### 3.4 Predictive performance of models

Regarding model performance, discrimination reported in development cohorts ranged from 0.66 to 0.996. All seven studies performed internal validation; one used a multicenter sample (23) and one used a nationwide sample (22). No external validation was reported. Among classification models that relied primarily on survey- and scale-based features, random forest (RF), artificial neural networks (ANN), and backpropagation neural networks (BPNN) generally achieved better discrimination than traditional logistic regression. In the study by Huiying Yang (19), the model yielded a higher AUC, and in the study by Man Liu et al. (20), BPNN outperformed logistic regression in both training and independent hold-out validation. In the study by Mamoudou Koume et al. (22), machine-learning models based on nationwide health insurance reimbursement data achieved a best AUC of 0.66 because of limited feature granularity and class imbalance; under other experimental settings, AUCs mostly fell within the moderate range of 0.49-0.66. Regarding calibration, four studies provided quantitative or graphical evidence: Huiying Yang (19) used the Hosmer-Lemeshow goodness-of-fit test; Yaru Li et al. (21) used both the Hosmer-Lemeshow test and a calibration curve; and Hui Ren (23) and Linlin Li (18) used calibration curves to assess agreement between observed and predicted outcomes. Performance of the risk prediction models is summarized in **Table 3**.

**Table 3:** Predictive performance of FCR models.

Author(s) (year)	AUC/C-index	AUC/C-index		Sensitivity		Specificity		Calibration	Verification method
		Build	Verify	Build	Verify	Build	Verify		
Man Liu et al.(2024) (20)	AUC	0.823	0.843	–	–	–	–	Not report	Internal
Yaru Li et al.(2024) (21)	AUC	0.976	0.89	–	–	–	–	Hosmer-Lemeshow test and calibration curve	Internal
Mamoudou Koume et al.(2024) (22)	AUC	0.66	–	0.79	–	–	–	Not report	Internal
Hui Ren et al.(2024) (23)	AUC	0.937	0.949	–	–	–	–	calibration curve	Internal
Linlin Li et al.(2025) (18)	C-index	0.996	–	–	–	–	–	calibration curve	Internal
Chao Fang et al.(2025) (17)	Not applicable								Internal
Huiying Yang (2022) (19)	AUC	–	Logistic:0.746 ANN:0.818 RF:0.892	–	Logistic:79.3% ANN:80.0% RF:89.4%	–	Logistic:86.9% ANN:75.8% RF:77.4%	Hosmer-Lemeshow test	Internal

AUC, area under the receiver-operating characteristic curve; C-index, concordance index; ANN, artificial neural network; RF, random forest; -,not reported.

### 3.5 Risk of bias and applicability

**Figure 2:**
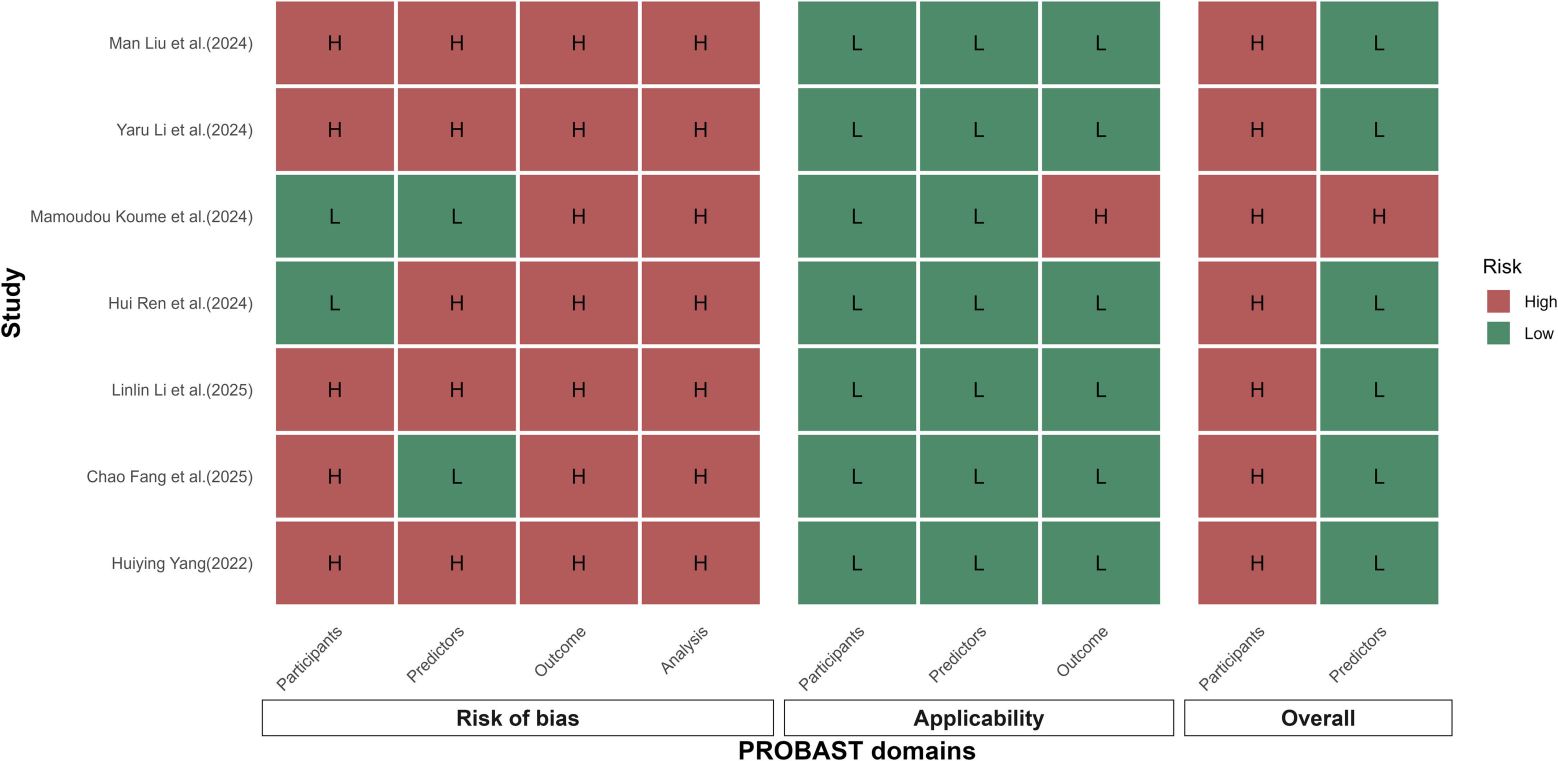
Risk-of-bias traffic-light plot.

All included studies showed good applicability across the participants, predictors, and outcome domains. However, the overall high risk of bias indicates methodological shortcomings in model development or validation. For risk of bias, in the participants domain, five studies (17–21) were judged at high risk, mainly because most were single-center and cross-sectional with limited sample representativeness, constraining applicability; the study by Mamoudou Koume et al. (22) used a nationwide sample and therefore had better representativeness. In the predictors domain, many studies relied on self-reported instruments (e.g., Social Support Rating Scale [SSRS], FoP-Q-SF), introducing potential information bias; several studies applied univariable/multivariable preselection or machine-learning-based importance ranking before modeling, with variable transparency. In the outcome domain, the definition of FCR was broadly consistent, but cutoff thresholds and instrument versions differed; a threshold of FoP-Q-SF ≥ 34 for “high FCR” was explicitly used in multiple studies, facilitating cross-study comparisons. In the analysis domain, only internal validation was performed, with no independent external validation; one study (19) reported an extremely high C-index which, together with a small sample and many predictors, suggested overfitting. Risk of bias and applicability assessments are presented in **Table 4**, **Figure 2**. In addition, we uploaded an example of the risk-of-bias assessment as Appendix 2.

**Table 4:** Results of bias and applicability risk assessment according to PROBAST.

Author(s) (year)	Risk of bias				Applicability			Overall	
	Participants	Predictors	Outcome	Analysis	Participants	Predictors	Outcome	Risk of bias	Applicability
Man Liu et al.(2024) (20)	H	H	H	H	L	L	L	H	L
Yaru Li et al.(2024) (21)	H	H	H	H	L	L	L	H	L
Mamoudou Koume et al.(2024) (22)	L	L	H	H	L	L	H	H	H
Hui Ren et al.(2024) (23)	L	H	H	H	L	L	L	H	L
Linlin Li et al.(2025) (18)	H	H	H	H	L	L	L	H	L
Chao Fang et al.(2025) (17)	H	L	H	H	L	L	L	H	L
Huiying Yang (2022) (19)	H	H	H	H	L	L	L	H	L

H, high risk of bias, high applicability risk; L, low risk of bias, low applicability risk.

## 4 Discussion

### 4.1 Effectiveness and risk-of-bias profile of prediction models for FCR in patients with cancer

Across the seven included studies, prediction models built from common psychosocial scales and readily available clinical variables showed good-to-excellent discrimination; some studies reported AUC or C-index values approaching or exceeding 0.95, suggesting strong within-sample separability in single-center or homogeneous cohorts. However, the PROBAST indicated that most studies relied on internal validation with limited external validation; transparency was also limited for handling of missing data, events-per-variable ratios, feature selection, and hyperparameter tuning. In addition, several studies reported strikingly high AUC/C-index values despite small-to-moderate sample sizes and minimal overfitting control or model parsimony procedures, raising concern for optimism bias. Regarding outcome measurement, most studies used the FCRI-SF or the FoP-Q-SF, but cutoff definitions varied (e.g., ≥13 for “high FCR” vs ≥22 for “clinical FCR”), and some mixed continuous and binary outcome definitions. These choices introduced outcome-domain heterogeneity, limiting comparability across models and affecting event-rate stability. With respect to populations and sampling, most studies were single-center and cross-sectional, with only a few multicenter or nationwide cohorts, which constrained representativeness and generalizability. Overall, current evidence supports the preliminary use of these models for local screening and risk stratification, whereas generalizability and robustness require confirmation through prospective, multicenter studies with rigorous external validation. In addition, the set and apparent importance of predictors varied across studies, which is expected given differences in cancer types, treatment contexts, and psychosocial measurement; this further underscores the need for standardized outcomes and external validation before transportability can be assumed.

The evidence base is also geographically concentrated. Six of the seven included studies were conducted in China, with only one conducted in France. This limits ethnic diversity and may reduce transportability of the models to other healthcare systems and cultural contexts. Because FCR appraisal, social support structures, and help-seeking behaviors differ across cultures, predictors and their coefficients may not generalize without recalibration. Future studies should prioritize cross-country, multi-ethnic prospective cohorts and perform external validation and recalibration, ideally with assessment of cross-cultural measurement equivalence for FCR instruments.

### 4.2 Predictive analysis of models for FCR in patients with cancer

In this study, common predictors of FCR clustered in the psychosocial domain, including fatigue, depression/anxiety, pain, social support/social constraints, cognition and metacognition, and emotion-regulation styles; in the demographic domain, variables such as age, income, and employment status also showed stable effects across several models. This aligns with the biopsychosocial nature of FCR (24, 25). In clinical practice, psychosocial indicators that are accessible, quantifiable, and modifiable should be prioritized, and patients who screen positive can be referred for psychological-behavioral interventions to support targeted supportive care. Methodologically, logistic regression paired with nomograms offers strong interpretability and point-of-care usability; machine-learning approaches such as random forest, gradient boosting, and deep/shallow neural networks are advantageous for capturing nonlinearity and interactions and are often used for feature selection and performance gains. However, when sample sizes are limited and predictors are highly correlated, the benefits of complex models may be constrained, and robust cross-validation, regularization, and model parsimony become essential to reduce overfitting and optimistic estimates. For clinical translation, visual nomograms, simplified scoring systems, and threshold-based risk stratification aid patient communication and intervention decisions; for high-risk groups requiring longitudinal monitoring, models can be embedded into follow-up systems, with decision curve analysis (DCA) used to appraise net benefit. Regarding data sources, exploratory models using “non-questionnaire” features such as insurance or prescription claims are feasible but currently show moderate discrimination, suggesting that these approaches may perform best when integrated with psychosocial scales or clinical questionnaires to achieve multimodal gains.

From an implementation standpoint, validated FCR instruments (e.g., FoP−Q−SF, FCRI−SF) remain the most practical tools for routine screening and symptom assessment. Prediction models should be framed as complementary tools for risk stratification and care planning, not direct replacements for FCR measurement. Future studies should test the incremental value of prediction models beyond existing scales—for example, gains in calibration, decision−curve net benefit, or reductions in assessment burden—using prospective multicenter cohorts, external validation, and transparent reporting aligned with TRIPOD.

### 4.3 Limitations of the systematic review process

This systematic review has several limitations. Although we searched nine major databases and screened reference lists, relevant studies may still have been missed due to incomplete indexing or non-inclusion in the searched sources. We included only Chinese- and English-language publications, which may introduce language bias. Because of substantial heterogeneity in outcome definitions, cutoff thresholds, predictor sets, and modeling/validation strategies, we did not perform a quantitative meta-analysis of model performance; consequently, our synthesis may be more susceptible to reporting bias in the primary studies. In addition, we did not contact original authors to clarify missing or unclear information, which may have led to more conservative judgments in PROBAST assessments. Most included models were derived from cross-sectional data, and external validation was limited, constraining robustness and transportability. Six of the included studies were conducted in China; therefore, applicability outside China should be interpreted with caution. Differences in healthcare pathways, follow-up schedules, insurance coverage, and psychosocial support systems across countries may shift baseline FCR prevalence and modify predictor effects, thereby affecting cross-setting performance.

### 4.4 Future directions and challenges for FCR prediction models in patients with cancer

With advances in artificial intelligence and machine learning, prediction modeling has progressed rapidly in recent years; however, modeling work specifically targeting FCR in patients with cancer started relatively late, and the validity and reproducibility of existing models remain inconsistent. Future studies should prioritize external validation and recalibration across institutions, cancer types, and cultural contexts; prospective, multicenter designs are preferred, with a clear separation of development, internal validation, and external validation phases, and complete reporting in accordance with TRIPOD and its extensions for machine-learning models. With respect to predictors and data sources, psychometric scales should be retained as core inputs while exploring multimodal integration with wearable-sensor data, medication/claims records, and simplified behavioral or linguistic features to improve generalizability while reducing participant burden. Although included studies used broadly consistent conceptual definitions of FCR, cutoff thresholds varied across instruments and settings. Future work should standardize measurement procedures, including cross-language and cross-cultural equivalence (measurement invariance) testing, and harmonize outcome definitions and thresholds; adequate sample size and events-per-variable should be ensured for complex models, and missing-data mechanisms should be explicitly characterized and handled using robust strategies. Collectively, these steps may improve predictive performance, enable fair model comparisons, and support more rigorous and transportable conclusions.

## 5 Conclusions

Evidence on prediction models for fear of cancer recurrence (FCR) in adults with cancer remains limited. Only seven eligible studies were identified, and several studies incompletely reported key FCR-related descriptive information and important modeling details. Although some models showed promising discrimination in development or internal validation, the overall risk of bias was high and external validation was largely lacking, which constrains robustness and generalizability. Therefore, these findings should be interpreted as preliminary and hypothesis-generating rather than practice-changing. Future research should prioritize prospective, multicenter studies with standardized FCR ascertainment and transparent reporting in accordance with TRIPOD and should perform rigorous external validation and recalibration (and, where appropriate, evaluate clinical utility using decision-curve analysis) before routine implementation.

## Data Availability

The original contributions presented in the study are included in the article/Supplementary Material. Further inquiries can be directed to the corresponding author.
