# Supplementary material for: Prediction models for fear of cancer recurrence in adults with cancer: a systematic review
Source: Front Oncol. 2026 Mar 19;16:1739251. doi: 10.3389/fonc.2026.1739251 (PMC13043336; doi:10.3389/fonc.2026.1739251)
Supplement: Supplementary file 1 [file DataSheet1.pdf]

Appendix 1. Search strategy

Database	Search strategy
PubMed	#1: “Neoplasms”[MeSH Terms]
	#2: “Neoplasms”[Title/Abstract] OR “Tumors”[Title/Abstract] OR “Neoplasia”[Title/Abstract] OR “Neoplasias”[Title/Abstract] OR “Neoplasm”[Title/Abstract] OR “Tumor”[Title/Abstract] OR “Cancer”[Title/Abstract] OR “Cancers”[Title/Abstract] OR “Malignant Neoplasm”[Title/Abstract] OR “Malignancy”[Title/Abstract] OR “Malignancies”[Title/Abstract] OR “Malignant Neoplasms”[Title/Abstract] OR “Neoplasm, Malignant”[Title/Abstract] OR “Neoplasms, Malignant”[Title/Abstract] OR “Benign Neoplasms”[Title/Abstract] OR “Neoplasms, Benign”[Title/Abstract] OR “Neoplasm, Benign”[Title/Abstract] OR “Benign Neoplasm”[Title/Abstract]
	#3: #1 OR #2
	#4: “fear of cancer recurrence”[Title/Abstract] OR “fear of recurrence”[Title/Abstract]
	#5: “prediction model”[Title/Abstract] OR “predictive models”[Title/Abstract] OR “risk prediction”[Title/Abstract] OR “risk assessment”[Title/Abstract] OR “risk score”[Title/Abstract]
	#6: #3 AND #4 AND #5
Embase	#1: 'neoplasm'/exp
	#2: neoplasm:ab,ti OR 'acral tumour':ab,ti OR 'acral tumor':ab,ti OR neoplasia:ab,ti OR neoplasms:ab,ti OR 'neoplastic disease':ab,ti OR 'neoplastic entity':ab,ti OR 'neoplastic mass':ab,ti OR tumor:ab,ti OR 'tumoral mass':ab,ti OR 'tumoral entity':ab,ti OR 'tumorous entity':ab,ti OR 'tumorous mass':ab,ti OR tumors :ab,ti OR tumour:ab,ti OR 'tumoural entity':ab,ti OR 'tumourous entity':ab,ti OR 'tumoural mass':ab,ti OR 'tumourous mass':ab,ti OR tumours:ab,ti

	#3: 'fear of cancer recurrence'/exp
	#4: 'fear of cancer recurrence':ab,ti OR 'cancer recurrence fear':ab,ti
	#5: 'predictive model'/exp
	#6: 'forecast model':ab,ti OR 'forecast modeling':ab,ti OR 'forecast modelling':ab,ti OR 'forecast simulation':ab,ti OR 'forecasting model':ab,ti OR 'prediction model':ab,ti OR 'predictive modeling':ab,ti OR 'predictive modelling':ab,ti OR 'predictive simulation':ab,ti OR 'predictive model':ab,ti
	#7: #1 OR #2
	#8: #3 OR #4
	#9: #5 OR #6
	#10: #7 AND #8 AND #9
Web of Science	TS =(Neoplasms OR Tumors OR Neoplasia OR Neoplasias OR Neoplasm OR Tumor OR Cancer OR Cancers OR Malignant Neoplasm OR Malignancy OR Malignancies OR Malignant Neoplasms OR Benign Neoplasms OR Benign Neoplasm) AND TS = (fear of cancer recurrence OR fear of recurrence) AND TS =(prediction model OR predictive models OR risk prediction OR risk assessment OR risk score)
Cochrane Library	#1: MeSH descriptor: [Neoplasms] explode all trees
	#2: (Cancer):ti,ab,kw OR (Malignant Neoplasms):ti,ab,kw OR (Neoplasm, Malignant):ti,ab,kw OR (Malignancies):ti,ab,kw OR (Cancers):ti,ab,kw
	#3: (Malignant Neoplasm):ti,ab,kw OR (Malignancy, Neoplasms):ti,ab,kw OR (Malignant):ti,ab,kw OR (Tumor):ti,ab,kw OR (Neoplasm):ti,ab,kw
	#4: (Tumors):ti,ab,kw OR (Neoplasias):ti,ab,kw OR (Neoplasia):ti,ab,kw OR (Neoplasm):ti,ab,kw OR (Benign):ti,ab,kw

	#5: (Benign Neoplasm):ti,ab,kw OR (Neoplasms, Benign):ti,ab,kw OR (Benign Neoplasms):ti,ab,kw
	#6: #1 OR #2 OR #3 OR #4 OR #5
	#7: (fear of cancer recurrence):ti,ab,kw OR (fear of recurrence):ti,ab,kw
	#8: (prediction model):ti,ab,kw OR (predictive models):ti,ab,kw OR (risk prediction):ti,ab,kw OR (risk assessment):ti,ab,kw OR (risk score):ti,ab,kw
	#9: #6 AND #7 AND #8
CINAHL	#1: “Neoplasms”[MeSH Terms]
	#2: “Neoplasms”[Title/Abstract] OR “Tumors”[Title/Abstract] OR “Neoplasia”[Title/Abstract] OR “Neoplasias”[Title/Abstract] OR “Neoplasm”[Title/Abstract] OR “Tumor”[Title/Abstract] OR “Cancer”[Title/Abstract] OR “Cancers”[Title/Abstract] OR “Malignant Neoplasm”[Title/Abstract] OR “Malignancy”[Title/Abstract] OR “Malignancies”[Title/Abstract] OR “Malignant Neoplasms”[Title/Abstract] OR “Neoplasm, Malignant”[Title/Abstract] OR “Neoplasms, Malignant”[Title/Abstract] OR “Benign Neoplasms”[Title/Abstract] OR “Neoplasms, Benign”[Title/Abstract] OR “Neoplasm, Benign”[Title/Abstract] OR “Benign Neoplasm”[Title/Abstract]
	#3: #1 OR #2
	#4: “fear of cancer recurrence”[Title/Abstract] OR “fear of recurrence”[Title/Abstract]
	#5: “prediction model”[Title/Abstract] OR “predictive models”[Title/Abstract] OR “risk prediction”[Title/Abstract] OR “risk assessment”[Title/Abstract] OR “risk score”[Title/Abstract]
	#6: #3 AND #4 AND #5

CNKI	SU=（“肿瘤”+“癌症”+“癌”） AND SU=（“复发恐惧”+“癌症复发恐惧”+“肿瘤复发恐惧”） AND SU=（“预测”+“模型”+“预测模型”+“风险预测”+“风险评估”+“危险因素”）
VIP	M = (肿瘤 OR 癌症 OR 癌) AND M =(癌症复发恐惧 OR 复发恐惧 OR 肿瘤复发恐惧) AND M = (预测 OR 预测模型 OR 模型 OR 风险预测 OR 风险评估 OR 危险因素)
Wanfang Data	主题:(“肿瘤” or “癌症” or “癌”) and 主题:(“复发恐惧” or “癌症复发恐惧” or “肿瘤复发恐惧”) and 主题:(“预测” or “模型” or “预测模型” or “风险预测” or “风险评估” or “危险因素”)
CBM	("预测"[全部字段:智能] OR "模型"[全部字段:智能] OR "预测模型"[全部字段:智能] OR "风险预测"[全部字段:智能] OR "风险评估"[全部字段:智能] OR "危险因素"[全部字段:智能]) AND ("复发恐惧"[全部字段:智能] OR "肿瘤复发恐惧"[全部字段:智能] OR "癌症复发恐惧"[全部字段:智能]) AND ("肿瘤"[全部字段:智能] OR "癌症"[全部字段:智能] OR "癌"[全部字段:智能])

Note: Because CNKI, VIP, Wanfang Data, and CBM are Chinese-language databases, the search strategies are retained in Chinese to facilitate reproducibility.
