# Supplementary material for: Prediction models for fear of cancer recurrence in adults with cancer: a systematic review
Source: Front Oncol. 2026 Mar 19;16:1739251. doi: 10.3389/fonc.2026.1739251 (PMC13043336; doi:10.3389/fonc.2026.1739251)
Supplement: Supplementary file 2 [file DataSheet2.pdf]

Appendix 2 an example of the risk-of-bias assessment

Using Mamoudou Koume's study—Predicting Fear of Breast Cancer Recurrence in Women Five Years After Diagnosis Using Machine Learning and Healthcare Reimbursement Data from the French Nationwide VICAN Survey—as an example, we will demonstrate how to assess risk of bias and applicability with PROBAST. The PROBAST judgments will follow the official guidance: "+" indicates low risk of bias / low concern regarding applicability, "–" indicates high risk of bias / high concern regarding applicability, and "?" indicates unclear risk of bias / unclear concern regarding applicability.For readability, in Table 4 ("Results of risk of bias and applicability assessment according to PROBAST"), we further define: H = high risk of bias / high applicability concern; L = low risk of bias / low applicability concern.

1. Participants

1.1 Were appropriate data sources used?

Judgement：+

Evidence: Nationwide VICAN-5 survey linked to reimbursement data, i.e., an appropriate cohort/survey source.

1.2 Were all inclusions and exclusions appropriate?

Judgement：?

Evidence: Eligibility and final n are described, but representativeness/attrition and missingness handling are not fully auditable in the visible text.

Participants—ROB：

With 1.1 positive and no explicit high-risk red flags demonstrated, you can classify Participants as low ROB (L) under your rule set.

Participants—Applicability：

Applicability low concern (L) is justified if your target population matches 5-year disease-free BC survivors as defined in the paper.

2. Predictors

2.1 Were predictors defined and assessed similarly for all participants?

Judgement：+

Evidence: Predictors were extracted from standardized reimbursement hierarchies applied consistently across participants.

2.2 Were predictor assessments made without knowledge of outcome data?

Judgement：?

Evidence: Potentially independent extraction, but no explicit statement of blinding/label isolation is visible.

2.3 Are all predictors available at the time the model is intended to be used?

Judgement：+

Evidence: Utilization/reimbursement predictors are available in the intended healthcare system setting.

Predictors—ROB：

With strong evidence for consistent definition and availability, and no demonstrated leakage, you can justify Predictors as low ROB (L).

Predictors—Applicability：

Low concern if your intended setting can access reimbursement/utilization data and accepts them as predictors.

3. Outcome

3.1 Was the outcome determined appropriately?

Judgement：−

Evidence: Single-item FCR and broad dichotomization increase misclassification/measurement bias; authors acknowledge bias risk.

3.2 Was a prespecified or standard outcome definition used?

Judgement：−

Evidence: Clear rule exists, but it is not a validated standard FCR scale definition/cutoff.

3.3 Were predictors excluded from the outcome definition?

Judgement：+

Evidence: Outcome is questionnaire-based, predictors are reimbursement-based; no incorporation.

3.4 Was the outcome defined and determined similarly for all participants?

Judgement：+

Evidence: Same question and dichotomization for all.

3.5 Was the outcome determined without knowledge of predictor information?

Judgement：?

Evidence: Likely independent but not explicitly stated.

3.6 Was the time interval between predictor assessment and outcome determination appropriate?

Judgement：?

Evidence: Predictor window definition relative to outcome timepoint not fully transparent.

Outcome—ROB：

Negative judgements on appropriateness/standardization with explicit author-acknowledged bias justify high ROB (H).

Outcome—Applicability：

High concern if your review expects validated scale-based FCR outcomes, not a single-item dichotomy.

4. Analysis

4.1 Reasonable number of participants with the outcome?

Judgement：?

Evidence: Sample/outcome counts reported, but model complexity vs events not fully specified.

4.2 Were continuous/categorical predictors handled appropriately?

Judgement：?

Evidence: Predictors include hierarchical categorical “classification levels” and

count/quantity-type variables, but the text does not describe encoding, transformations, or standardization
4.3 Were all enrolled participants included in the analysis?
Judgement：+
Evidence: The flow diagram/population description shows 918 participants included for modeling, and no additional exclusions are described for the analysis set.
4.4 Were participants with missing data handled appropriately?
Judgement：−
Evidence: Exclusions due to missing medical data are noted, but missing-data handling strategy is not described, a key PROBAST concern.
4.5 Was selection of predictors based on univariable analysis avoided?
Judgement：?
Evidence: Multiple feature-selection methods are reported (e.g., SFM/RFE/ReliefF), but it is not stated whether any prior univariable screening was used, so avoidance of univariable selection cannot be confirmed.
4.6 Were complexities accounted for appropriately?
Judgement：?
Evidence: The outcome is binary (no censoring/time-to-event modeling), and the authors note they used counts of events without incorporating timing; any survey sampling structure is not clearly modeled/adjusted in the described analysis, leaving uncertainty about handling of such complexities.
4.7 Were relevant model performance measures evaluated appropriately?
Judgement：−
Evidence: Discrimination metrics reported, but calibration/external validation not evident; generalizability needs further evaluation.
4.8 Were overfitting/optimism accounted for?
Judgement：−
Evidence: Resampling + feature selection + model selection without clearly described nested/locked validation implies high optimism risk.
4.9 Do predictors and their weights correspond to the reported multivariable analysis?
Judgement：?
Evidence: The paper provides feature-importance/SHAP-style interpretation, but does not present a fully specified final model with explicit coefficients/weights (or a deployable equation/parameter set) in the visible text, so correspondence to the multivariable model cannot be verified.

Analysis—ROB：

Negative judgements on missing data handling, performance evaluation (calibration/external validation), and optimism control justify high ROB in Analysis.

Overall ROB = H

Outcome and Analysis high ROB lead to overall high ROB (worst-domain rule).

Overall Applicability = H

High concern in Outcome applicability drives overall high concern.
